# Accumulation of Bisphenol A^®^ by *Pleurotus* spp.—Flow Injection Analysis

**DOI:** 10.3390/molecules29112520

**Published:** 2024-05-27

**Authors:** Agata Krakowska, Małgorzata Suchanek, Robert Piech, Beata Paczosa-Bator, Tomasz Skalski, Bożena Muszyńska

**Affiliations:** 1 Department of Inorganic Chemistry and Pharmaceutical Analytics, Faculty of Pharmacy, Jagiellonian University Medical College, 9 Medyczna Street, 30-688 Kraków, Poland; 2 Department of Analytical Chemistry and Biochemistry, Faculty of Materials Science and Ceramics, AGH University of Krakow, Al. Mickiewicza 30, 30-059 Krakow, Poland; msuchanek@agh.edu.pl (M.S.); paczosa@agh.edu.pl (B.P.-B.); 3 Tunneling Group, Biotechnology Center, Silesian University of Technology, Bolesława Krzywoustego 8, 44-100 Gliwice, Poland; tomasz.skalski@polsl.pl; 4 Department of Pharmaceutical Botany, Faculty of Pharmacy, Jagiellonian University Medical College, 9 Medyczna Street, 30-688 Kraków, Poland; muchon@poczta.fm

**Keywords:** accumulation, adsorption, desorption, bisphenol A^®^, *Pleurotus* spp., extraction, artificial digestive juices, flow injection analysis

## Abstract

A specific feature of mushrooms (including those of the genus *Pleurotus*) is their natural ability to absorb and accumulate many chemical substances present in their immediate environment, which makes them an excellent natural sorption material. Hence, fruiting bodies of mushrooms have been recognized for years as excellent indicators of the environment, reflecting its current state. Nevertheless, mushrooms can accumulate both health-promoting substances, such as bioelements, and toxic substances, such as heavy metals and organic compounds, including bisphenol A^®^ (BPA). This organic chemical compound in the phenol group, although it has been withdrawn in the EU since 2010, is widely present in the environment around us. In the present experiment, we aimed to determine the effect of adding BPA to liquid media for in vitro cultures of *Pleurotus* spp. The biomass increases were determined. Moreover, the degrees of adsorption and desorption of BPA from the obtained freeze-dried biomass in two different environments (neutral and acidic) were determined as a function of time. This is the first study to determine the bioavailability of adsorbed BPA in obtained biomass by extracting the mycelium into artificial digestive juices in a model digestive system. BPA was added to the liquid Oddoux medium in the following amounts: 0.01, 0.5, and 0.5 g/250 mL of medium. The amounts of adsorbed and desorbed BPA were determined by flow injection analysis (FIA) with amperometric detection. The addition of BPA to the substrate reduced the biomass growth in each of the discussed cases. BPA adsorption by the mycelium occurred at over 90% and depended on the morphology of the mushroom (structure, surface development, and pore size). BPA desorption depended on the pH of the environment and the desorption time. Mushrooms are an excellent natural remedial material, but BPA is extracted into artificial digestive juices; therefore, consuming mushrooms from industrialized areas may have health consequences for our bodies.

## 1. Introduction

The specific ability of higher mushrooms, including *Pleurotus* species, to accumulate has been the subject of numerous studies and scientific publications, especially in recent years [1,2,3,4,5,6,7]. The first reports on bioaccumulation concerning the sorption of some metals by mushrooms appeared nearly 100 years ago [8,9]. Initially, the focus was on assessing the toxicity of mushrooms resulting from the accumulation of heavy metals. A little later, the practical possibilities of using mushrooms as natural dietary ingredients enriched with selected substances began to be noticed [10,11,12,13]. Research on the specific properties of mushrooms gained importance after World War II, when attention began to be paid to their extraordinary features, which include their natural ability to effectively absorb and accumulate all chemical entities present in their immediate environment. Hence, fruiting bodies of “wild” mushrooms have been recognized for years as excellent environmental bioindicators [14,15,16,17] and used for mycoremediation. The uptake of substances by mushrooms is closely related to their availability in the substrate [18,19,20]. The presented work focused on the use of mycelial mushrooms as a natural material with specific sorption properties for bisphenol A^®^. This organic chemical compound in the phenol group was one of the most widely produced chemicals in the world [21], which is why it is widely distributed in the environment, even though it has been withdrawn in the European Union since 2010 [22]. It was used in the production of plastics, including polycarbonate, polyesters (present in baby feeding bottles and thermal papers for printers) [23,24,25], polysulfones, and epoxy resins, used for lining metal cans for canning and storing food, toys, drinking containers, sports equipment, medical equipment, and dental monomers, among others. Moreover, it was used in the food industry as an antioxidant and in cheaper cosmetics [23]. Studies have shown that despite its useful properties, this compound is toxic. It causes inflammation and numerous diseases of the genital organs (the ovaries, prostate, and endometrium [26]) and breasts. It has also been proven that BPA has an adverse effect on human reproduction by disturbing the functioning of the ovaries and their functioning during in vitro fertilization [27], deteriorating the properties of semen [28], and hindering the implantation of embryos [29]. Additionally, high levels of BPA in the body disturb the functioning of the thyroid gland [30,31]. Therefore, in the presented work, research was carried out on the accumulation in mycelial mushrooms of this dangerous compound for humans, which is still widely present in our environment. For this purpose, in vitro cultures of three species of mushrooms of the *Pleurotus* species (*P. djamor*, *P. pulmonarius*, and *P. ostreatus*) were established in media with the addition of bisphenol A^®^. There were several reasons for selecting these species for this research. These species belong to saprotrophic woody mushrooms, and unlike mycorrhizal mushrooms, they are much more active in the decomposition of chemical substances (including toxic substances) due to their high content of lignocellulolytic enzymes. Moreover, the specific structure of the hyphae of these mushrooms means that they have already been used, for example, to remove heavy metals, including Cd, Pb, or As [32]. Additionally, these mushrooms are obtained from crops and have a great taste and numerous health-promoting properties for humans (containing minerals, carbohydrates, phenolic and indole compounds [33], and acidic polysaccharides [34]). The influence of the addition of bisphenol A^®^ in various amounts (0.05, 0.1, and 0.5 g of BPA/250 mL of medium) on its accumulation in the mycelium and biomass growth was examined. The efficiency of its adsorption and the degree of leaching of this compound depending on the time under the influence of two environmental factors (water and acid precipitation) were determined. Moreover, the influence of the mushroom’s structural morphology (surface development, size, and number of pores) on the degree of BPA accumulation was determined (using SEM and BET analysis). BPA, which is a component of many plastics, is still present in the environment due to the fact that the decomposition of plastics is a long-term process. Therefore, alternative activities to support this process are being sought. One of the effective solutions is the use of biodegradation. Therefore, the conducted research aimed to demonstrate the possibility of using mycelium as a natural material for the accumulation of BPA. However, at the stage after the sorption of BPA by the mycelium, this material would require disposal because BPA is desorbed from it. Therefore, its consumption would pose a health risk to humans. This was confirmed by our research, in which the obtained biomasses of the tested mushroom species were extracted into artificial gastric and intestinal digestive juices in a model digestive system. This made it possible to determine the degree of risk (the amount of bisphenol A^®^) posed by the entry of this dangerous substance into the body when consumed in mushroom material. The analysis was carried out using the flow injection analysis (FIA) method. The obtained results broaden the knowledge of BPA accumulation by the mycelium of mushrooms and may contribute to the development of elimination and biodegradation methods for BPA.

## 2. Materials and Methods

### 2.1. The Scheme of the Experiment

**Figure 1:**
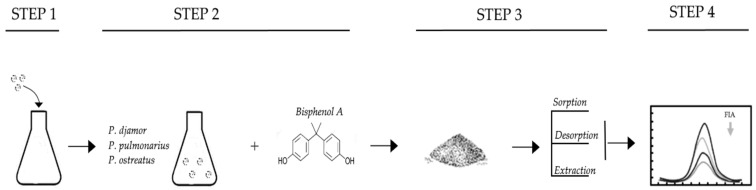
Scheme of the conducted experiment.

This research was carried out according to the scheme presented in Figure 1.

### 2.2. Mushroom Materials

#### 2.2.1. Initial Mycelial Cultures (Step 1)

Mycelial cultures of three species of mushrooms of the *Pleurotus* genus were selected for the experiment: *Pleurotus djamor* (Rumph. ex Fr.), *Pleurotus ostreatus* (Jacq.), and *Pleurotus pulmonarius* (Fr.), which were obtained from an agar medium. The obtained biomasses from the in vitro cultures on solid media were transferred in an amount of 0.1 g of the inoculum into 500 mL Erlenmeyer flasks filled with 250 mL of a liquid medium (Oddoux medium (1957)). The in vitro cultures were performed while maintaining physicochemical conditions that were optimized in previous experiments [35]. Representative samples of the mycelia were deposited in the Department of Pharmaceutical Botany of the Jagiellonian University Medical College.

#### 2.2.2. Experimental Mycelial Cultures (Step 2)

The experimental materials consisted of *Pleurotus* species biomasses obtained in the liquid Oddoux medium with the addition of bisphenol A^®^ in an amount of 0.05, 0.1, or 0.5 g/250 mL of the medium (0.88 mM, 1.75 m, or 8.76 mM). The weighed amounts of bisphenol A^®^ were dissolved in 1 mL of methanol using an ultrasonic bath (45 min) (Emag Emmi 20 HC, Mörfelden-Walldorf, Germany) and then quantitatively transferred to flasks.

In vitro shaken cultures were performed in 500 mL Erlenmeyer flasks with three independent replicates. After 14 days of mycelial growth, the mushroom material obtained from each tested species was separated from the medium by filtration (Pyrex Buchner funnel with a perforated plate, Merck, Darmstadt, Germany). The obtained biomass was freeze-dried (Freezone 4.5 freeze dryer, Labconco, Kansas, MO, USA; temperature: −40 °C).

### 2.3. Mushroom Material Lyophilization Analysis (Step 3)

#### 2.3.1. Sorption and Desorption

The obtained freeze-dried biomasses of the three *Pleurotus* species (step 1) from the experiment were subjected to a sorption and desorption study for bisphenol A^®^ in order to determine the degree of its connection with the matrix. The degree of sorption was determined by analyzing the difference between the content of bisphenol A^®^ in the initial medium for the shaken mycelial cultures to which it was added in an amount of 0.05, 0.1, or 0.5 g/250 mL of the medium (0.88 mM, 1.75 mM, or 8.76 mM) and its amount remaining in the medium after 14 days of culture. In turn, in order to determine the degree of desorption, amounts of 0.5 g of the obtained biomasses were transferred to 50 mL of a solution. Desorption tests were carried out in two environments: an aqueous environment and that corresponding to acid rain conditions (using a 10% nitric acid solution, for which 65% nitric acid was diluted in quadruple-distilled water). The analysis was performed in three independent repetitions each time.

#### 2.3.2. Extraction of Bisphenol A^®^ into Artificial Digestive Juices (Step 3)

##### Preparation of Artificial Digestive Juices

In order to carry out the planned experiment, solutions of artificial digestive juices (saliva, gastric juice, and intestinal juice) were prepared. The individual ingredients of the digestive juices are presented in Table 1. All ingredients were weighed with an accuracy of 0.1 mg with an analytical scale. The components of the individual digestive juices weighed in this way were transferred to flasks with a volume of 1000 mL, which were filled to the mark with quadruple-distilled water.

##### Extraction Process

The obtained biomasses (step 2) were homogenized with an analytical mill (EGK, Rommelsbacher, Plauen, Germany). The materials thus obtained were weighed to an amount of 0.5 g and extracted in the artificial digestive juices. The process was carried out in three independent repetitions. For this purpose, the weighed amounts of the biomasses were placed in flat-bottomed flasks. Then, the mushroom materials were moistened with 3 mL of artificial saliva. An amount of 20 mL of artificial gastric juice was added to the materials. The mixtures prepared in this way were incubated for 60 min in a Gastroel-2014 apparatus (Krakow, Poland) [39]. This device allows extraction tests to be carried out in physicochemical conditions similar to those prevailing in the human body (37 °C). After a period of 1 h, the contents of the flat-bottomed flasks were separated from the filtrates by filtration using membrane paper (Ø 0.22 µm, Millex, Millipore Corporation, Burlingtone, MA, USA). In the next step, 20 mL of intestinal juice was added to the separated biomasses, and the digestion process was continued for 150 min so as to maintain the natural sequence of the digestion process and the time corresponding to the physiological period of stay of food content in the human digestive system. After this period, the filtration process was repeated. The bisphenol A^®^ content was determined in the obtained filtrates using the FIA method.

### 2.4. Reagents

In the presented experiment, the bisphenol A^®^ standard was purchased from Merck (Darmstadt, Germany, cat. no.: 80057). Cultures: ZnCl_2_ (cat. no.: 229997) and MgCl_2_ (cat. no.: 63138) were purchased from Sigma-Aldrich (Darmstadt, Germany). The ingredients of the artificial digestive juices: NaCl and NaHCO_3_ were obtained from PPH Golpharm (Kraków, Poland); pepsin and bile salts were obtained from BTL (Łódź, Poland); CaCl_2_ was obtained from Pharma Zentrale GmbH (Herdecke, Germany); pancreatic extract, HCl, KCl, concentrated HNO_3_, Suprapur^®^, and KNO_3_ were purchased from Merck (Darmstad, Germany); and C_6_H_8_O_7_, KHCO_3_, Na_2_HPO_4_, K_2_HPO_4_, KH_2_PO_4_, and NaOH were acquired from the Polish Society of Chemistry (Gliwice, Poland). The HPLC-grade methanol used to dissolve bisphenol A^®^ was purchased from Sigma-Aldrich (St. Louis, MO, USA). The quadruple-distilled water used with a conductivity of less than 1 µS/cm was obtained using an S2-97A2 distillation apparatus (ChemLand, Stargard Szczecin, Poland).

### 2.5. Analytical Tools Applied

In the presented experimental work on the mycoremediation of bisphenol A^®^ by the mycelia of the Pleurotus species, the degree of adsorption, desorption, and extraction of bisphenol A^®^ into plastic digestive juices was determined using the measurement technique of FIA. The visualization of the characteristics and surface development of the materials (the mycelia of the Pleurotus species) was performed using scanning electron microscopy (SEM) and the Brunauer–Emmett–Teller isotherm (BET).

#### 2.5.1. Surface Characteristics

The microstructures and surface morphologies of the *P. djamor*, *P. pulmonarius*, and *P. ostreatus* mycelia (the homogenized biomasses from the in vitro cultures) were observed under an SEM microscope (FEI Nova NanoSem 200, Thermo Fisher Scientific, Hillsboro, OR, USA) with an accelerating voltage of 18 kV and magnification of 3.000×. The surface areas and total pore volumes of the three mycelia (*P. djamor*, *P. pulmonarius*, and *P. ostreatus*) were measured by N2 adsorption using the Brunauer–Emmett–Teller (BET) and Langmuir methods (ASAP 2010, Micromeritics, Norcross, GA, USA).

#### 2.5.2. Bisphenol A^®^ Content Analysis (FIA Detection) (Step 4)

**Figure 2:**
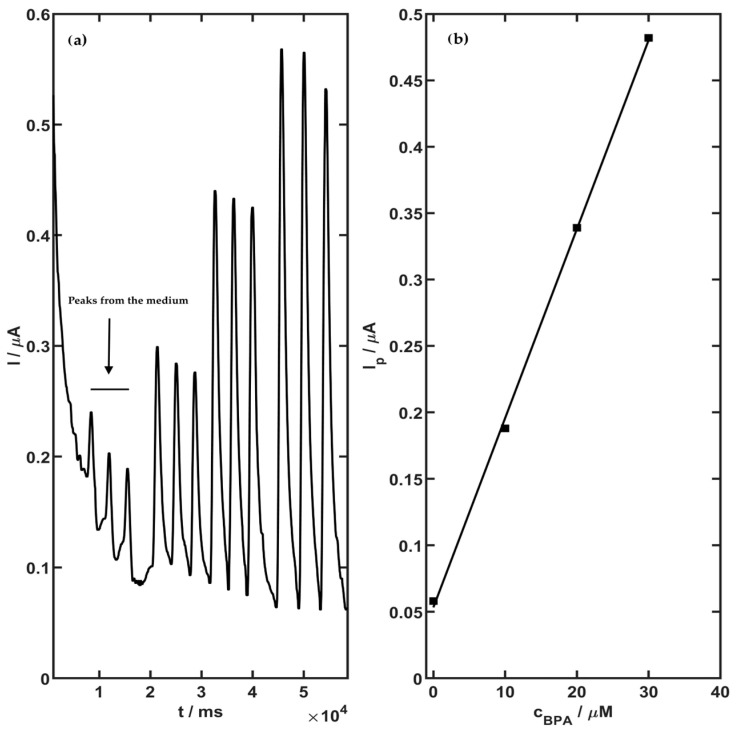
2,2-bis (p-hydroksyfenylo)propan calibration chart (**a**) and curve (**b**) for concentration range from 10 to 30 µM using screen-printed carbon electrodes with amperometric parameters of measurements under flow injection conditions.

The flow system was composed of a 0.05 L K_2_HPO_4_ (0.01 M) solution reservoir and an 800 Dosino pump connected to a 900 Touch Control panel (Metrohm, Herisau, Switzerland), as well as a sample injection valve (Rheodyne Model 7010), a flow wall-jet detector, and a valve with a sample loop with a volume of 100 µLA. Screen-printed carbon electrodes (SPCEs) with built-in reference electrodes (Metrohm, Herisau, Switzerland) were used as well. Amperometric determination of BPA under flow injection conditions was performed. The characteristic parameters (working potential and flow rate) of the FIA were investigated. The optimal potential was chosen as 700 mV, and the calibrations were registered with an optimal flow rate of 0.01 M K_2_HPO_4_ equal to 1.0 mL/min. The signals for BPA determination under flow conditions were registered in a BPA concentration range of 10 to 30 µM. The BPA concentration in the medium was determined under flow analysis conditions. The measurements were carried out using the calibration method. The calibration curves were registered in 0.01 M K_2_HPO_4_ containing 50 µL of the medium. The calibration curve for BPA determination in the range of 10 to 30 µM by FIA is presented in Figure 2. The obtained slope of the regression line for BPA was 0.0142 ± 0.003 µA/µM (intercept: 0.0533 ± 0.0051 µM; r = 0.9997).

## 3. Results and Discussion

In the presented research work, the degree of adsorption of bisphenol A^®^ dosed into the substrate (culture medium) was analyzed for three amounts (0.05, 0.1, and 0.5 g/250 mL of medium) depending on the species of the *Pleurotus* mycelia (*P. djamor*, *P. ostreatus*, and *P. pulmonarius*). In turn, the obtained biomasses were subjected to desorption analysis, which was carried out in two environments (neutral and acidic) at three time points (1 h, 2 h, and 6 h), and extraction in artificial digestive juices. The obtained natural materials (biomasses from in vitro cultivation) were characterized. The results are presented in the following subsections.

### 3.1. Material Characteristics

**Figure 3:**
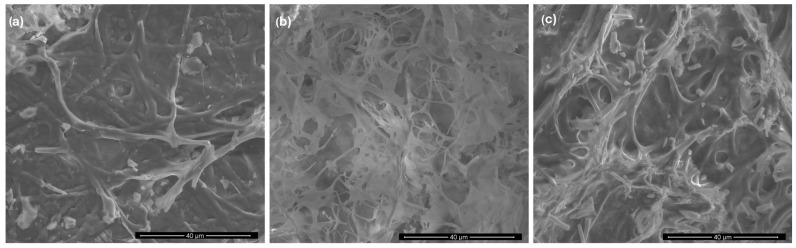
SEM images of *P. djamor* (**a**), *P. pulmonarius* (**b**), and *P. ostreatus* (**c**) mycelia.

The SEM images of the *P. djamor*, *P. pulmonarius*, and *P. ostreatus* mycelia are shown in Figure 3. As can be seen, the *P. ostreatus* mycelium has an obvious microporous structure, which can provide a high specific surface area. The least porous structure is observed for the *P. djamor* mycelium, which can provide a low specific surface area.

By means of the BET method, it was found that the specific surface areas of the examined mycelia were 1.47 m^2^/g for *P. djamor,* 1.92 m^2^/g for *P. pulmonarius*, and 4.21 m^2^/g for *P. ostreatus*. The surface areas obtained by the Langmuir equation were 2.19 m^2^/g, 2.82 m^2^/g, and 6.58 m^2^/g for the *P. djamor, P. pulmonarius*, and *P. ostreatus* mycelia, respectively. The total micropore volumes of *P. djamor*, *P. pulmonarius*, and *P. ostreatus* were 0.25 mm^3^/g, 0.197 mm^3^/g, and 1.094 mm^3^/g, respectively. The average pore diameters of the mycelia were 13.6 nm, 12.0 nm, and 12.1 nm for *P. djamor*, *P. pulmonarius*, and *P. ostreatus*, respectively.

### 3.2. Biomass Growth Analysis

**Figure 4:**
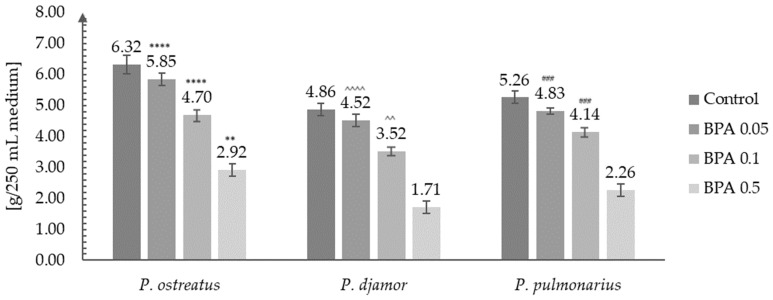
Biomasses obtained after in vitro cultures of *Pleurotus* species (*P. djamor, P. ostreatus,* and *P. pulmonarius*) in the liquid Oddoux medium (g/250 mL of medium) depending on the amount of bisphenol A^®^ added (0.05, 0.1, or 0.5 g/250 mL of medium) (one-way ANOVA with post hoc Tukey’s test: ** *p* < 0.01 and **** *p* < 0.0001 vs. *P. djamor* control; ^^ *p* < 0.01 and ^^^^ *p* < 0.0001 vs. *p. pulmonarius* control; ### *p* < 0.001 vs. *P. ostreatus* control).

The analysis of biomass growth was performed on freeze-dried and homogenized materials. For this purpose, the biomass obtained from the three independent cultures for each species and the addition of bisphenol A^®^ was weighed with an analytical scale with an accuracy of four decimal places. The results are presented in Figure 4.

The analysis showed that the addition of bisphenol A^®^ to the medium, even in a small amount, reduced the growth of the mycelial biomass (Figure 4). The highest increase in biomass for the smallest addition of bisphenol A^®^ (0.05 g/250 mL of medium) was obtained in the case of the in vitro culture of the *P. ostreatus* species (5.85 g of dry biomass/250 mL of medium). In turn, the lowest biomass, in this case, was observed in the in vitro cultures of the *P. djamor* species. The lowest biomass increase was recorded for the addition of 0.5 g of BPA to the medium. In this case, an over 50% decrease in biomass was observed for all *Pleurotus* species discussed in this work (Figure 4). Despite the observed decreases in biomass growth for the in vitro cultures of the *Pleurotus* species obtained in media with the addition of BPA, their values were still higher than, for example, in the case of the *P. pulmonarius* species obtained by Włodarczyk et al. in 2021 conducted in a medium enriched with MgSO_4_·7H_2_O salts (6.58 g/L of medium) (control: 2.89 g/L of medium) [35].

Similarly, in another experiment (Confortin et al., 2008) in which the production of mycelial biomass was optimized, a maximum of 5.49 g of mycelium per liter of substrate was obtained in the case of *Pleurotus* sajor-caju [40]. In turn, in other experiments in which the researchers added different concentrations of glucose to the media (Rosado et al., 2003), they achieved a higher biomass growth efficiency, which, in the extreme case, amounted to as much as 22.8 g of dry mycelium per 1 L of the medium [41,42].

### 3.3. Sorption Analysis

**Figure 5:**
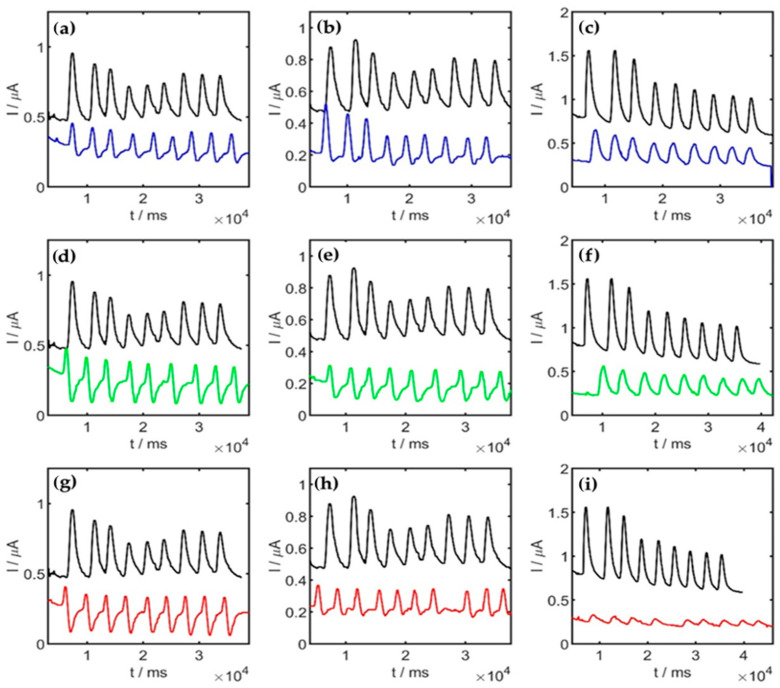
BPA signals registered in medium before (black) and after sorption of 0.05 g (**a**,**d**,**g**), 0.1 g (**b**,**e**,**h**), and 0.5 g (**c**,**f**,**i**) of BPA by *P. djamor* (blue), *P. pulmonarius* (green), and *P. ostreatus* (red).

Flow injection analysis (FIA) with amperometric detection was used for the BPA determination in the medium before and after sorption. The operating parameters are given in Section 2.5.2. The FIA signal was recorded each time in three independent repetitions with the addition of the medium in the range of 5 to 50 µL. The obtained FIA curves for the medium before and after sorption by *P. djamor*, *P. pulmonarius*, and *P. ostreatus* are presented in Figure 5. The BPA concentration in the medium was calculated from the regression line using the following equation:

I_p = 0.0143∙c_BPA + 0.0533

Based on the sorption analysis, it was shown that mushrooms of the *Pleurotus* species effectively sorbed bisphenol A^®^ from the substrate in each of the discussed cases (Table 2).

The analysis of the sorption efficiency showed that its degree decreased with the increase in the amount of BPA added to the medium. In the case of the addition of 0.05 g of BPA/250 mL of medium, the sorption efficiency for all species was over 90%. In turn, in the case of the addition of the highest concentration—0.5 g of BPA/250 mL of medium—a large disproportion was observed (Table 2). The species distinguished by the highest sorption efficiency was *P. ostreatus* (over 90% sorption). In turn, the lowest sorption efficiency was demonstrated by the *P. djamor* mycelium (65.3% sorption). This indicates that the sorption efficiency is related to the morphology of the mycelium, including its structure, surface development, size, and the shape of its pores. The species with the highest surface area and pore size and number had the best sorption, i.e., *P. ostreatus* (Section 3.1).

The tests carried out on the accumulation of BPA by mycelium show that this material is an excellent sorbent and can be an alternative to existing solutions. So far, BPA degradation has mainly involved recycling plastics, in which BPA is returned to the environmental cycle. Hence, the use of biodegradation is a more effective way of degrading plastics due to the environmentally friendly mechanism that reduces their pollution. Moreover, this process can be carried out under controlled in vitro conditions, as shown in this work. Mushrooms play a key role in the biodegradation of plastics by secreting degrading enzymes, i.e., cutinase, lipase, proteases, and lignocellulolytic enzymes. The action of these enzymes is based on the effective hydrolysis or oxidation of polymers, resulting in the formation of functional groups that improve their hydrophilic properties and, as a result, cause their decomposition. So far, several species of mushrooms are known whose degradative effect has been confirmed, e.g., *Aspergillus* species and *Cladosporium* species. These also include saprotrophic mushrooms, e.g., *Agaricus bisporus* and *Pleurotus* spp., in particular, *Pleurotus ostreatus*, which was the subject of the above work [43].

### 3.4. Desorption Analysis

**Figure 6:**
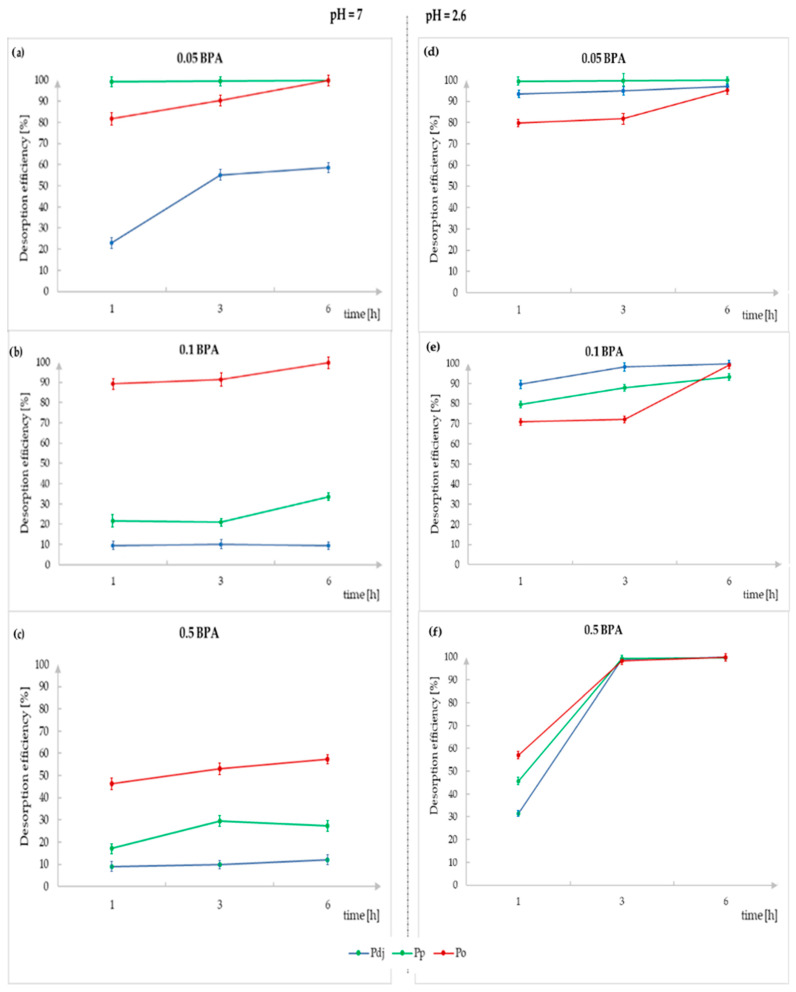
A graph presenting the desorption of BPA in a neutral environment with pH = 7 and acidic environment with pH = 2.6 for *Pleurotus* species mycelia (*P. djamor* (blue line), *P. pulmonarius* (green line), and *P. ostreatus* (red line)) depending on the desorption time (1, 3, or 6 h) and BPA additive to the medium (**a**,**d**—00.5, **b**,**e**—0.1, or **c**,**f**—0.5 g of BPA/250 mL of medium).

In order to determine whether the bisphenol A^®^ absorbed from the substrate during mycelial growth undergoes the reverse process, desorption, the obtained freeze-dried biomass was weighed to an amount of 0.5 g and, in three independent repetitions, introduced into 50 mL of a solution of quadruply distilled water (pH = 7) and an acidic solution, corresponding to acid precipitation in the natural environment (pH = 2.6). Desorption was carried out at three time points, 1, 3, and 6 h, after which samples were taken and analyzed using the FIA method. The obtained results are presented in Figure 6.

#### 3.4.1. Desorption in Water

The analysis of BPA desorption in an aqueous environment (pH = 7) showed that for the mycelia obtained in a medium with the addition of 0.05 g of BPA/250 mL of medium, *P. djamor* was desorbed to the lowest degree (58.6%), and *P. pulmonarius* and *P. ostreatus* were desorbed to the highest and comparable degree (over 90%). Moreover, in the case of the first species mentioned, the degree of desorption increased with time. In turn, for the *P. pulmonarius* and *P. ostreatus* species, the amount of desorbed BPA stabilized after 3 h (Figure 6). However, in the case of the analysis of BPA desorption from the mycelia obtained in a medium with the addition of 0.1 g of BPA/250 mL of medium, a decrease in the desorption efficiency was observed for the *P. djamor* species (desorption after 6 h: 9.4%) and the *P. pulmonarius* species (desorption after 6 h: 33.3%). Only in the case of *P. ostreatus*, the decrease in desorption was small and amounted to approximately 1%. A completely different effect was observed in the case of the *P. ostreatus* mycelium obtained in a medium with the addition of 0.5 g of BPA/250 mL of medium. In this case, the degree of desorption dropped by over 40%, and after 6 h, it was 57.3%. Additionally, it was observed that the desorption carried out in an aqueous environment stabilized after 3 h, and the further desorption did not significantly increase its degree.

#### 3.4.2. Desorption in Acidic Media

**Figure 7:**
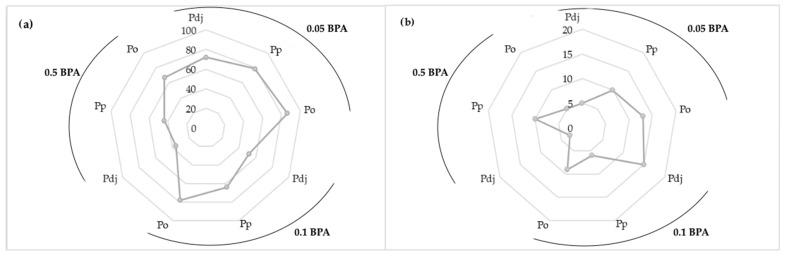
Graph showing the desorption of BPA into artificial digestive juices—(**a**) gastric juice and (**b**) intestinal juice (Pdj—*P. djamor*, Pp—*P. pulmonarius*, and Po—*P. ostreatus*)—from the biomass obtained from breeding with the addition of 0.05, 0.1, and 0.5 g/250 mL of BPA to the medium.

The study of BPA desorption in an acidic environment (pH = 2.6) showed that the pH significantly affected the desorption efficiency. In this case, desorption occurred to a higher degree for all of the discussed *Pleurotus* species. The degree of desorption increased to over 80% for the *P. djamor and P. pulmonarius* species in the case of the analysis of the desorption from the biomass obtained in a culture with the addition of BPA at a level of 0.1 g/250 mL of medium and up to over 90% in the case of adding 0.5 g/250 mL of medium. In turn, for the *P. ostreatus* species, the desorption from the biomass obtained for all three BPA additives was at a similar level and amounted to over 90%. Also, in the case of desorption into an acidic environment, as well as into a neutral-pH environment, the desorption time depended on the degree of desorption. Also, in this case, the degree of desorption stabilized after 3 h, and further incubation in the solution did not significantly increase its effectiveness (Figure 7b).

### 3.5. Extraction into Artificial Digestive Juices

The efficiency of bisphenol A^®^ extraction from the obtained biomasses from the in vitro cultures of the *Pleurotus* species depending on the extraction site (gastric juice or intestinal juice) is presented in Figure 7a,b.

Based on the extraction into artificial digestive juices, it was found that the degree of extraction depended on the place of extraction (gastric juice or intestinal juice) as well as on the species of mushroom (*P. djamor*, *P. pulmonarius*, or *P. ostreatus*). The highest extraction efficiency was observed in artificial gastric juice, at over 80%). This was a similar trend to that in the case of desorption in an acidic medium. In that case, the pH in the gastric juice was also acidic and was approximately two. Additionally, this reaction affected the destruction of the mycelial cell walls, which resulted in a larger amount of BPA released from the inside of the mycelium. The highest extraction efficiency was observed in the case of the *P. ostreatus* species, and it was 83% in the gastric juice solution (Figure 7a) and 11% in the intestinal juice (Figure 7b). However, extraction by the *P. djamor* and *P. pulmonarius* species occurred to a similar extent and was at a level of 45%. The extraction in the intestinal juice, the pH of which was slightly alkaline (pH = 8), ranged from 3 to 15%. The low level of extraction in this place was also influenced by the fact that most of the BPA from the mycelia was extracted earlier in the gastric juice. Although BPA is an organic substance, similar to the analysis of metals in mushrooms, its extraction into gastric juice is more effective, which is related to the pH that occurs in the gastric juice, which is strongly acidic [44].

## 4. Conclusions

The conducted research confirms that mycelial mushrooms accumulate chemical entities present in their environment (e.g., toxic bisphenol A^®^). Hence, they constitute a natural material with adsorption properties. Even though BPA has been withdrawn from use, it is still present in the environment around us. The efficiency of BPA adsorption is closely related to the morphology of the mushrooms, i.e., their surface development and the number and size of pores. In turn, the degree of desorption depends on the pH of the solution and is much greater in the case of solutions with a pH below seven, as demonstrated in this work. Moreover, it was shown that this compound was extracted from mushroom biomass into artificial digestive juices to a degree of over 80%, so consuming mushrooms from industrialized areas poses a potential threat to human health and life. Studies have shown that mycelium is a good material for the accumulation of BPA, but whether its mechanism is physisorption or chemisorption has not been specified. In the next stage, research work will be aimed at explaining this, which will enable us, in the future, to propose an effective method of eliminating BPA from the material and, consequently, reducing it in the natural environment.

## Figures and Tables

**Table 1:** Compositions of artificial digestive juices [36,37,38].

	**Chemical Compounds**	**Weight (g)/L**	Direction of extraction 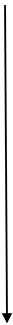
Saliva (pH = 6.7)	CaCl_2_	0.20	
	C_6_H_8_O_7_	0.03	
	KHCO_3_	1.50	
	KH_2_PO_4_	0.35	
	MgCl_2_	0.01	
	Na_2_HPO_4_	0.35	
Gastric juice (pH = 2)	HCl	0.10	
	NaCl	2.00	
	Pepsin	3.20	
Intestinal juice (pH = 8)	Bile salt	0.15	
	NaHCO_3_	8.50	
	Pancreatic extract	0.02	

**Table 2:** Sorption efficiencies (shortcuts: *P. djamor*—Pdj, *P. pulmonarius*—Pp, and *P. ostreatus*—Po).

	Sorption of 0.05 g of BPA/250 mL of medium [%]
Pdj	92.8 ± 6.4
Pp	95.9 ± 3.1
Po	**99.1** ± 0.8
	Sorption of 0.1 g of BPA/250 mL of medium [%]
Pdj	91.3 ± 1.5
Pp	93.3 ± 1.2
Po	**99.0** ± 0.6
	Sorption of 0.5 g of BPA/250 mL of medium [%]
Pdj	65.3 ± 7.3
Pp	66.9 ± 4.6
Po	**97.5** ± 1.3

## Data Availability

Data are contained within the article.
